# A Case of Idiopathic Intracranial Hypertension Complicated with both Infratentorial and Supratentorial Cortical Superficial Siderosis: Novel Imaging Findings on Intravoxel Incoherent Motion Magnetic Resonance Imaging Offering Clues to Pathophysiology

**DOI:** 10.3390/neurolint16040053

**Published:** 2024-06-28

**Authors:** Shinya Watanabe, Yasushi Shibata, Eiichi Ishikawa

**Affiliations:** 1 Department of Neurosurgery, Mito Kyodo General Hospital, Tsukuba University Hospital Mito Area Medical Education Center, Mito, 3-2-7 Miyamachi, Ibaraki 310-0015, Japan; yshibata@md.tsukuba.ac.jp; 2 Department of Neurosurgery, Institute of Medicine, University of Tsukuba, 2-1-1, Amakubo, Tsukuba, Ibaraki 305-8576, Japan; e-ishikawa@md.tsukuba.ac.jp

**Keywords:** idiopathic intracranial hypertension, superficial siderosis, migraine, papilledema, ocular abduction disorder, intravoxel incoherent motion, magnetic resonance imaging, headache

## Abstract

The pathology of idiopathic intracranial hypertension (IIH), a disease characterized by papillary edema and increased intracranial pressure (IICP), is not yet understood; this disease significantly affects quality of life due to symptoms including vision loss, headache, and pulsatile tinnitus. By contrast, superficial siderosis (SS), a disorder in which hemosiderin is deposited on the surface of the cerebral cortex and cerebellum, potentially causes cerebellar ataxia or hearing loss. So far, no cases of IIH with infratentorial and supratentorial cortical SS have been reported. Herein, we report a case of a 31-year-old woman with obesity who developed this condition. The patient suddenly developed headache and dizziness, had difficulty walking, and subsequently became aware of diplopia. Fundus examination revealed bilateral optic nerve congestive papillae and right eye abducens disturbance. Head magnetic resonance imaging (MRI) showed prominent SS on the cerebellar surface and cerebral cortex. Lumbar puncture revealed IICP of 32 cmH_2_O, consistent with the diagnostic criteria for IIH, and treatment with oral acetazolamide was started; subsequently, the intracranial pressure decreased to 20 cmH_2_O. Her abduction disorder disappeared, and the swelling of the optic papilla improved. She was now able return to her life as a teacher without any sequelae. SS is caused by persistent slight hemorrhage into the subarachnoid space. In this case, both infratentorial and supratentorial cortical superficial SS was observed. Although cases of IIH complicated by SS are rare, it should be kept in mind that a causal relationship between IIH and SS was inferred from our case. Our findings also suggest that cerebrospinal fluid dynamic analysis using MRI is effective in diagnosing IIH and in determining the efficacy of treatment.

## 1. Introduction

Idiopathic intracranial hypertension (IIH) is characterized by increased intracranial pressure as indicated by papilledema and imaging findings, with no identifiable extrinsic causes [1]. The main symptoms are headache, vision loss, and pulsatile tinnitus, which are known to significantly affect quality of life and visual function [1]. The cause is unknown but is thought to involve overproduction of cerebrospinal fluid (CSF), absorption disorders, impaired venous return, and metabolic disorders [2]. The incidence is known to be higher in obese women of reproductive age [3] at approximately 5.5 per 100,000 per year [4], while the reported overall incidence is approximately 1 to 2 per 100,000 per year [4,5,6]. The overarching goal of treatment is to alleviate symptoms and prevent permanent vision loss. It has been reported that IIH can also be treated by treating obesity [7]. On the other hand, superficial siderosis (SS) is a disorder in which hemosiderin is deposited on the cortical surface of the cerebrum or cerebellum, and although hemorrhagic tendency, tumors, trauma, and spinal disease are classic known hemorrhagic factors, the cause of SS is also frequently unknown [8]. Persistent hemorrhage owing to spinal cord dural defects resulting from injury has also been implicated [9] and categorized as “duropathy” [10,11]. An association between spontaneous intracranial hypotension (SIH) and SS has also been reported [12,13]. However, so far, no cases of IIH with infratentorial and supratentorial cortical SS have been reported.

We encountered a very rare case of IIH complicated by infratentorial and supratentorial cortical SS. We aimed to report this case because of its clinical significance, as we uncovered novel findings in a patient with IIH that could aid in elucidating its pathology.

## 2. Case Description

This report was approved by the Institutional Review Board of Mito Kyodo General Hospital and was registered under the clinical trial registration number, IRB NO 23-09. We obtained written informed consent from the patient for reporting this case.

**Figure 1:**
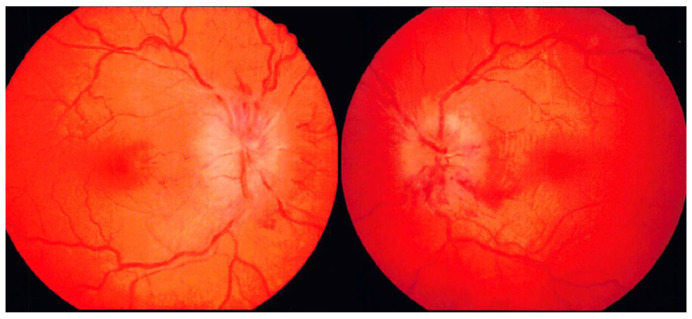
Congestive papillary findings in idiopathic intracranial hypertension. Fundus findings showed significant swelling of both optic nerve papillae.

A 31-year-old woman with obesity developed sudden headache and dizziness 13 days before hospital admission, following which vomiting continued for 3 days. Next, she lost strength in her legs and had difficulty walking. Subsequently, her headache, nausea, and dizziness, which peaked during the aforementioned 3 days, gradually subsided. Seven days before her admission, she became aware of diplopia when she turned her head to the right side. On the day of emergent admission, she visited an ophthalmologist at a nearby clinic and was referred to our hospital because of congestive papillae in both eyes and marked abduction disorder in the right eye. She also experienced dizziness during eye movements. On visiting our hospital, the patient was unable to walk independently. She did not smoke, drank alcohol only once per week, and had no history of allergies. She was Irish, had never been married or given birth, and was a middle school English teacher. She had a medical history of migraine with aura since puberty. Her most recent oral medications included eletriptan hydrobromide, lomerizine hydrochloride, domperidone, and herbal remedies for migraine. She had been on the pill until approximately 1.5 months ago but had stopped taking it on the advice of her family doctor due to an increased risk of stroke. She had no notable family history. Physical examination revealed obesity, with a height of 162 cm, weight of 80 kg, and body mass index of 30.5. Her awareness score was 15 (4-5-6) points on the Glasgow Coma Scale. On ophthalmologic examination, corrected visual acuity was 1.5 in both the right and left eyes, and intraocular pressure was not elevated (right eye: 13.5 mmHg, left eye: 13.0 mmHg); however, fundus findings showed significant swelling of both optic nerve papillae, which were stasis-like (Figure 1).

The cranial nervous system assessment revealed the following: prompt pupillary counter-reflexes, pupillary size of 3.0 mm, abducent nerve disturbances in the right eye, and absence of facial paralysis and dysarthria. An assessment of the motor system revealed a negative Barre’s sign and grade of 5/V on the Manual Motor Test in both the upper and lower limbs. No obvious sensory disturbances were observed in the whole body. The coordination of hand rotation was smooth. Her blood data showed no abnormalities in blood counts or general biochemistry and no hyperlipidemia or diabetes mellitus, and D-dimer levels were not elevated in the coagulation system.

**Figure 2:**
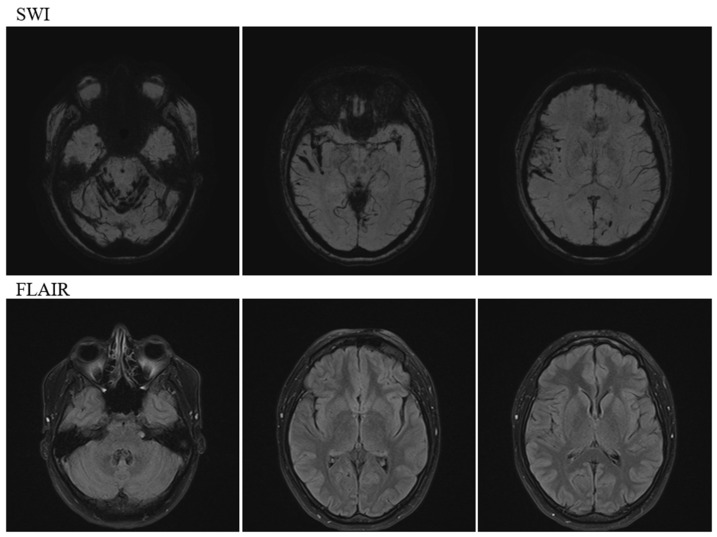
Magnetic resonance imaging on admission. SWI showing low intensity findings on the cerebellar hemispheric surfaces and cranial nerves under the cerebellar tent and low intensity findings on cortical surfaces, such as the Sylvian fissure on the tent. FLAIR imaging showing no occupying lesion and no obvious abnormalities. SWI: susceptibility-weighted imaging, FLAIR: fluid attenuated inversion recovery.

Head magnetic resonance imaging (MRI) (Figure 2) revealed multiple prominent hemosiderin deposits on the cerebellar surface and similar hemosiderin deposits in the temporal lobe and fissure sylviae, indicating infratentorial and cortical SS.

**Figure 3:**
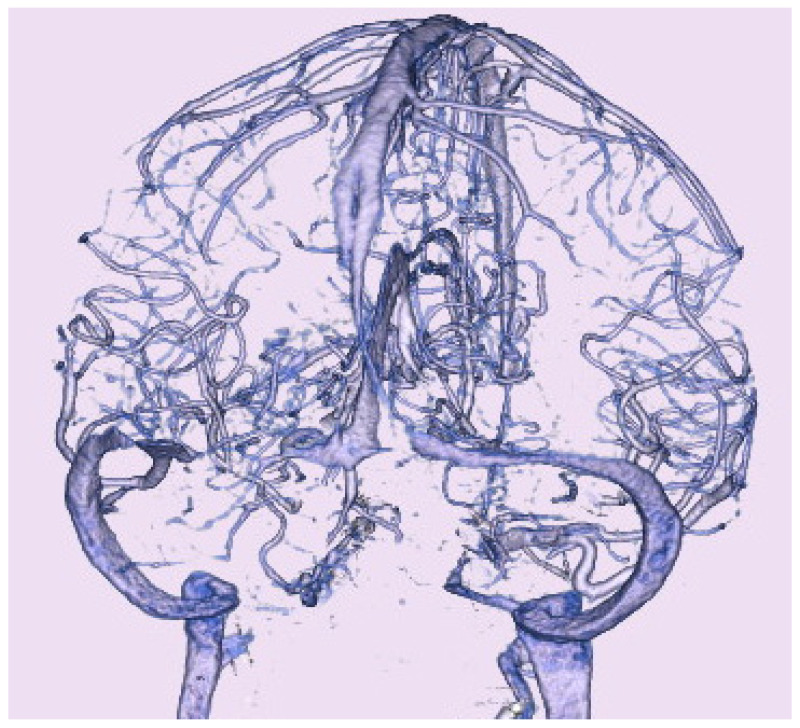
Computed tomography venography on admission. Computed tomography venography showing bilateral transverse sinus stenosis findings on maximum intensity projection.

Computed tomography (CT) showed no acute hemorrhage. CT venography (Figure 3) showed bilateral stenosis of the transverse sinus or bilateral dilatation of the adjoining vein of Labbe.

Lumbar puncture on day 1 after her admission showed an increased intracranial pressure of 32 cmH_2_O, and the following were the laboratory findings in the CSF sample: colorless and transparent CSF, specific gravity of 1.006 (normal range: 1.005–1.007), glucose level of 78 mg/dL (normal range: 40–75), protein level of 25 mg/dL (normal range: 10–40), and cell count of 4/3. Additional laboratory tests showed no notable findings. Therefore, she was diagnosed with IIH because no other disease that could cause her symptoms was apparent, and there was no obvious cause of intracranial hypertension, such as an occupying lesion, which aligned with the modified Dandy criteria [14] for the diagnosis of IIH. Treatment with oral acetazolamide (750 mg/day) was started. Approximately 3 days after treatment initiation, the abduction disorder in the patient’s right eye almost disappeared, her dizziness disappeared, and she was able to walk unassisted. Ten days from admission, the patient was discharged on her own. Twenty-two days from admission, a follow-up lumbar puncture showed a decreased intracranial pressure of 20 cmH_2_O, and the following were the laboratory findings in the CSF sample: colorless and transparent CSF, specific gravity of 1.006 (normal range: 1.005–1.007), glucose level of 68 mg/dL (normal range: 40–75), protein level of 40 mg/dL (normal range: 10–40), and cell count of 5/3. Sixty-three days from admission, on examination by the ophthalmologist, all findings had improved. Dynamic visual field testing showed that although the enlargement of Mariotte’s blind spot was still present and not yet normalized, it had shrunk. Ocular position testing showed that the strabismus and eye movement disorder had disappeared. Fundus examination showed that the swelling of the optic nerve papilla had improved, with only slight residual swelling left, and the subjective complaints of diplopia were no longer present. Seventy-one days from admission, head MRI showed no change in intracranial SS, and spinal MRI showed no CSF leakage that could be a cause of infratentorial siderosis. All of her aftereffects had disappeared, as evidenced by a modified Rankin scale score of 0, and she returned to her pre-onset life as a teacher.

**Figure 4:**
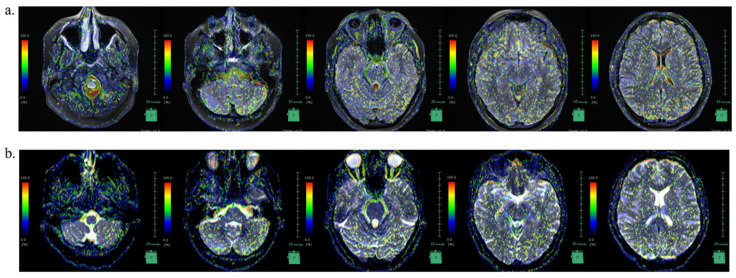
IVIM MRI findings showing the intracranial hypertension state and intracranial pressure reduction with acetazolamide administration. (**a**). IVIM MRI in the intracranial hypertension state before oral acetazolamide treatment, (**b**). IVIM MRI showing decreased intracranial pressure after oral acetazolamide treatment. IVIM MRI: intravoxel incoherent motion magnetic resonance imaging.

Intravoxel incoherent motion (IVIM) MRI, which was used to visualize CSF movement [15], using the IVIM Map (FUJIFILM Corporation), showed (Figure 4) that the SS site matched the area of strong incoherent CSF motion at the time of pre-treatment in our case as well.

In the IICP condition, incoherent CSF movement was pronounced in the caudal portion of the medulla oblongata, fourth ventricle, lateral ventricles, and brain surface; however, in the decreased intracranial pressure condition following acetazolamide treatment, the incoherent movement in these regions decreased (Table 1).

## 3. Discussion

In this case, a young woman with obesity and IIH developed sudden onset headache and nausea, followed by right eye abduction disorder and papilledema. The patient had marked SS in both the infratent and supratent. There have been no previous reports of IIH patients with both infratentorial and supratentorial SS. IVIM MRI was performed to visualize the movement of the CSF (clinical trial registration number: Mito Kyodo General Hospital IRB No. 23-09), suggesting a causal relationship between IIH and SS. Therefore, this report is highly novel and of high clinical significance as it provides insights into the relationship between IIH and SS and has revealed novel imaging findings, which could be clues regarding the pathophysiology.

### 3.1. Causal Relationship between IIH and SS

SS of the central nervous system is characterized by linear hemosiderin deposition in the superficial layers of the leptomeninges, cerebrum, and spinal cord [16]. Subarachnoid space infratentorial SS is thought to be caused by recurrent or continuous slight hemorrhage into the subarachnoid space, with the most important cause being spinal dural lesions that cause CSF leakage [16]. The classical types of infratentorial SS have been reported to involve dural abnormalities, spinal CSF leakage, craniocervical surgery, spinal trauma, head trauma, arteriovenous malformation (AVM), and tumors, while the secondary types involve subarachnoid hemorrhage (SAH), AVM, and cerebral venous embolism due to ruptured cerebral aneurysm [16]. The other types of SS have been classified into SS confined to the supratentorial compartment or cortical SS. Supratentorial cortical SS involves cerebral amyloid angiopathy in the elderly, ruptured cerebral aneurysmal SAH, AVM, reversible cerebral vasoconstriction syndrome, amyloid-related imaging abnormalities due to hemosiderin deposition, vasculitis, severe stenosis of the cerebral vessels, sinus occlusion, head trauma, and tumors [16].

In the present case, hemosiderin deposition was observed above and below the tent. However, there was no obvious cause of SS. There were no findings in the CSF specimen that would suggest persistent hemorrhage due to CSF leakage, as described in classical SS; similarly, head and spinal cord MRI findings did not reveal the presence of any disease that could have caused SS. However, the possibility that IIH may have caused the onset of SS due to arachnoid damage could not be ruled out although this has not been previously reported. Therefore, it should be kept in mind that IIH may cause SS due to arachnoid membrane rupture, which is a newly identified cause of SS.

Conversely, to the best of our knowledge, there are no reports regarding the possibility of IIH caused by SS which have documented the mechanisms of IIH, such as CSF malabsorption associated with SS. However, it is important to accumulate case reports similar to ours in the future.

### 3.2. Was SS Asymptomatic in This Case?

Typical neurological manifestations of infratentorial SS include slowly progressive sensorineural hearing impairment and cerebellar manifestations, such as ataxia, motor tremor, nystagmus, and dysarthria [8,9,17,18,19,20,21,22]. There are reported cases in which hemosiderin deposition in the cranial nerves has caused the appearance of individual cranial nerve symptoms [23].

The patient’s main symptoms of congestive papillae and abducens nerve palsy disappeared due to a successful response to acetazolamide and decrease in intracranial pressure; therefore, we believe that these symptoms were not caused by SS but by IIH. However, it is undeniable that the abducens nerve palsy in our patient at the time of intracranial hypertension onset may have been influenced by hemosiderin deposition in the cranial nerves.

### 3.3. Did SS Affect the Clinical Course of IIH?

In cerebral surface hemosiderin deposition disease, hemosiderin in the blood is deposited on the surface of the brain and spinal cord due to persistent and repeated bleeding, causing nerve damage due to oxidative stress [24]. At the time of admission, there were no acute hemorrhagic changes that could have resulted in a high signal on CT, and we believe that hemosiderin deposition was originally part of the patient’s previous history. MRI at the time of admission also showed hemosiderin deposition in the abducens nerve; however, we cannot rule out the possibility that neuropathy caused by oxidative stress may have precipitated the appearance of abducens nerve palsy due to IICP, although not to the extent that it caused continuous subjective symptoms.

### 3.4. CSF Circulation in IIH with SS

We were not able to find reports on CSF dynamics in IIH in the literature. Recently, IVIM MRI was used to visualize CSF movement [15]. In this case, the SS site matched the area of strong incoherent CSF motion at the time of pre-treatment. Our finding of incoherent CSF movement supports the association between IIH and SS.

The combination of SS and IIH has not been previously reported. However, SIH could cause SS by a CSF leak [12,13]. There were some reports of SIH converting to IIH by spontaneous closure of the leak. Sulioti et al. reported a case of a patient with IIH developing SIH and concluded that the implication of a possible causative link between elevated CSF pressure and subsequent development of dural rupture and SIH raises important questions regarding the pathophysiology of SIH in some cases [25]. Some reports revealed that increased craniospinal elastance can lead to elevated ICP, which is pathological in IIH and compensatory in SIH [26,27]. Parikh et al. reported on spontaneous decompression from underlying IIH as a possible etiology of SIH [28]. Similar to the findings in these previous cases, it is not impossible that the patient in our case had potential SIH and associated SS and developed IIH with the natural closure of the leak.

### 3.5. Limitations

This study had the following limitation. IVIM MRI may be subject to susceptibility artifacts; however, we confined the measurement area to a much smaller region of interest when performing the value measurements as a countermeasure to overcome this concern.

## 4. Conclusions

This is the first reported case of IIH with underlying cerebral surface hemosiderin deposition both above and below the tent; there was no obvious condition, such as CSF leakage, which could have caused SS, suggesting a causal relationship between IIH and SS. CSF analysis using IVIM MRI may be effective for diagnosing IIH and determining treatment efficacy.

## Figures and Tables

**Table 1:** Mean f values (%) in IVIM MRI in the IICP state before treatment and decreased ICP state after treatment.

	IICP State	Decreased ICP State
Foramen magnum	70.6	86.9
Right foramen of Luschka	44.3	89.1
Left foramen of Luschka	49.2	91.9
Foramen of Magendie	96.1	90.4
Medullary cistern	75.5	52.6
Fourth ventricle	87.4	77.3
Right cerebellopontine angle	11.7	97.7
Left cerebellopontine angle	96.8	59.7
Right inferior horn of lateral ventricles	11.5	10.1
Left inferior horn of lateral ventricles	4.1	6.8
Interpeduncular cistern	66.1	34.4
Subarachnoid space of right temporal lobe	94.8	13.1
Subarachnoid space of left temporal lobe	33.3	22.1
Right sylvian fissure	93.8	4.1
Left sylvian fissure	97.3	4.8
Third ventricle	80.8	57.5
Right anterior horn of lateral ventricles	98.6	9.6
Left anterior horn of lateral ventricles	1	98.9
Right foramen of Monro	58.3	3.5
Left foramen of Monro	96.2	1.3
Right body of lateral ventricles	89.9	47
Left body of lateral ventricles	99.2	0.9
Subarachnoid space of right frontal lobe	94.3	47
Subarachnoid space of left frontal lobe	98.7	98.2
Right central sulcus	86.7	90.8
Left central sulcus	94.4	53.6
Subarachnoid space of right parietal lobe	69	96.5
Subarachnoid space of left parietal lobe	83.5	96

f-value: Quantitative value of the subcomponent of microperfusion in IVIM MRI expressed as a numerical value from 0 to 100%. IVIM MRI: intravoxel incoherent motion magnetic resonance imaging, IICP: increased intracranial pressure, ICP: intracranial pressure

## Data Availability

The original contributions presented in the study are included in the article, and further inquiries can be directed to the corresponding author.
